# Malaria in Children

**DOI:** 10.4084/MJHID.2012.073

**Published:** 2012-11-06

**Authors:** Richard-Fabian Schumacher, Elena Spinelli

**Affiliations:** Ospedale dei Bambini, Children’s University Hospital, A.O. Spedali Civili, Brescia, Italy

## Abstract

This review is focused on childhood specific aspects of malaria, especially in resource-poor settings. We summarise the actual knowledge in the field of epidemiology, clinical presentation, diagnosis, management and prevention.

These aspects are important as malaria is responsible for almost a quarter of all child death in sub-Saharan Africa. Malaria control is thus one key intervention to reduce childhood mortality, especially as malaria is also an important risk factor for other severe infections, namely bacteraemia.

In children symptoms are more varied and often mimic other common childhood illness, particularly gastroenteritis, meningitis/encephalitis, or pneumonia. Fever is the key symptom, but the characteristic regular tertian and quartan patterns are rarely observed. There are no pathognomonic features for severe malaria in this age group. The well known clinical (fever, impaired consciousness, seizures, vomiting, respiratory distress) and laboratory (severe anaemia, thrombocytopenia, hypoglycaemia, metabolic acidosis, and hyperlactataemia) features of severe falciparum malaria in children, are equally typical for severe sepsis.

Appropriate therapy (considering species, resistance patterns and individual patient factors) – possibly a drug combination of an artemisinin derivative with a long-acting antimalarial drug - reduces treatment duration to only three days and should be urgently started.

While waiting for the results of ongoing vaccine trials, all effort should be made to better implement other malaria-control measures like the use of treated bed-nets, repellents and new chemoprophylaxis regimens.

## Epidemiology

The epidemiology of malaria in children is difficult to assess as most of clinical symptoms are non-specific and most of the cases occur in settings where no routine testing is available.

Malaria remains a leading cause of ill health. More than 40% of the world's population (approximately 3 billion people) are exposed to malaria in 108 endemic countries. It caused between 655 000 and 1.240.000^1^ deaths in 2010. Approximately 81% of malaria cases and 91% of malaria deaths occur in the African Region, where it remains one of the commonest causes of death and serious morbidity, especially for children and pregnant women; approximately 86% of malaria deaths globally are of children under 5 years of age.^2^ In fact children are at highest risk for severe disease and death between six months and five years of age: during this period children are most vulnerable as they have lost maternal immunity and they haven't yet developed specific immunity to infection. However this does not mean that younger infants are exempt from the death toll, the contrary is true given the fact that in addition to the well known inoculum through the blood meal of an infected female anopheles and through infusion of infected blood products, neonates and young infants might also be vertically infected by plasmodia crossing the placenta.

All taken together this makes the infection with the species *Plasmodium falciparum* one of the leading causes of child death from infectious diseases worldwide, according to new data it may be responsible for up to 24% of total child death in sub-Saharan Africa.^1^ This region, where falciparum is the predominant Plasmodium species, is in fact the home of almost all population at medium and high risk. In areas of high stable transmission, morbidity and mortality are highest in young children in whom acquired protective immunity is insufficient to protect against severe disease. Areas of low or unstable transmission are subject to malaria epidemics, and people of all ages are at risk of severe disease. There is substantial geographical overlap between malaria and HIV, and co-infection is associated with increases in parasite density and case fatality.

The epidemiology of falciparum malaria has been changing over the past 10 years, with declining numbers of clinical cases reported in different parts of the world. In Africa, malaria deaths have been cut by one third within the last decade; outside of Africa, 35 out of the 53 countries affected by malaria, have reduced cases by 50% in the same time period. In countries where access to malaria control interventions has improved most significantly, overall child mortality rates have fallen by approximately 20%,^3^ a percentage more than twice that of all childhood death attributable to malaria. Part of this reduction may be due to the fact now recognized that malaria is also an important risk factor for other severe infections, namely bacteraemia in African children.^4^

Use of artemisinin based combination therapies (ACTs) and increased coverage with insecticide-treated nets and indoor residual spraying have undoubtedly contributed to the falling number of cases. This improvement has been associated with a change in the observed age pattern of clinical malaria: in costal Kenya the mean age of children admitted to hospital with a positive malaria blood slide has increased from 3 years to 5 years.^5^,^6^

*P.vivax* is the most prevalent of the five human malaria parasites outside Africa. It is mostly absent from central and west Africa because a high proportion of the population have the Duffy-negative phenotype, which prevents erythrocyte invasion by the parasite. In other tropical regions of the world, *P.vivax* coexists with other Plasmodium species and mixed infections are common. Because transmission rates are low in most regions where *P.vivax* is prevalent, affected population do not achieve high levels of immunity to the parasite and people of all ages are at risk of infection, although children are more often ill.^6^ The increasing evidence that *P.vivax* is getting more and more chloroquine resistant in Asia^7^ is particularly important in the light of the fact that this highly transmissible Plasmodium species is at least as dangerous as the falciparum, especially to infants.^8^

## Clinical Features

The clinical manifestations of malaria, the severity and course of a clinical attack depends on the species and strain of the infecting plasmodium parasite, as well as the age, genetic constitution (ethnicity),^9^ immune status, malaria specific immunity, and nutritional status of the child, the mode of transmission of infection, whether the individual was on prophylaxis or had previous exposure to antimalarial drugs, as the latter may present with only minimal symptoms or signs.

The malaria paroxysm results from the lysis of parasitized red blood cells and release of merozoites into the circulation at the completion of asexual reproduction. The paroxysm is characterized by fever and chills accompanied by constitutional symptoms, alternating with periods of fatigue but otherwise relative wellness. Although periodicity of the paroxysm in primary attacks is thought to be pathognomonic for malaria species, this periodicity may take several days to become established, may not occur at all in asynchronous infections, or may be modified by previous immunity or treatment. In patients with previous malaria who are partially immune, merozoite release by erithrocytic schizonts and the accompanying febrile paroxysms are synchronous: approximately every 48 hours for *P.vivax* and *P.ovale* and every 72 hours for *P.malariae. P.falciparum* infections are usually asynchronous, resulting in nonperiodic febrile episodes, at least during the first days of illness.^10^

In children symptoms are varied and often mimic other common childhood illness particularly gastroenteritis, meningitis/encephalitis, or pneumonia. Fever and headache may be the sole symptoms, or gastrointestinal symptoms may predominate. Fever is the key symptom, but the characteristic regular tertian and quartan patterns are seen in < 25% of children; however, children are more likely to have high fever (>40°C), which may also lead to febrile convulsions. Nausea and vomiting are also common (especially for *P.falciparum*) and may hamper treatment with oral anti-malaria drugs.^11^ Pneumonia and acute diarrhea are the most common comorbid conditions associated with malaria and are both strong predictors of mortality. The diagnosis of pneumonia in a child with malaria might be a coexisting bacterial or viral respiratory illness, but the diagnosis might also be given to a child with malaria-related respiratory distress. Likewise, acute diarrhoea might be a feature of clinical malaria, or the result of concurrent diarrheal disease from an enteric pathogen.

Even if rigors frequently accompany infections with *P.vivax*, compared with adults, children are less likely to complain chills, arthralgia/myalgia or headache but are more likely to have hepatomegaly, splenomegaly and jaundice.^12^

In general, severity of symptoms and risk of death increase with increasing parasitaemia.

*P.falciparum* malaria is the most severe form of malaria, with fatality rates up to 15% in non-immune children with anaemia and severe respiratory distress if appropriate therapy is not promptly instituted. Since *P.falciparum* is the only Plasmodium species that infects all ages of erythrocytes, it can lead to intense parasitaemia that can reach 60% or more. Malaria caused by the other species of Plasmodium usually results in parasitaemias of less than 2%. *P.vivax* and *P.ovale* preferentially infect reticulocytes, and *P.malariae* infects mostly senescent red cells. Thus, severe complications of malaria are more often encountered in *P.falciparum* infection.

However, only a small proportion of the large number of people infected with *P.falciparum* develop severe malaria.

### Complications

Many studies have attempted to decipher which aspects lead malaria infection to severe disease in some, yet remain asymptomatic in others [13]. The likelihood of death is increased in children with pre-existing health problems such as anaemia, malnutrition and immunocompromised states. Asplenic patients develop rapidly progressive malaria.

Malaria complications result from haemolytic anaemia and microvascular obstruction with subsequent tissue ischemia. Features of severe or complicated malaria include respiratory distress, acidosis (pH <7.3), hypoglycaemia (<2.2 mmol/l), elevated aminotransferases, severe anaemia (Hb <5 g/dl), and high parasitaemia (defined as >5%–10% infected erythrocytes or more than 500 000 infected erythrocytes per microliter).

It is important to remember that there are no clinical features that are pathognomonic for severe malaria. The well known clinical (fever, impaired consciousness, seizures, vomiting, respiratory distress) and laboratory (severe anaemia, thrombocytopenia, hypoglycaemia, metabolic acidosis, and hyperlactataemia) features of severe falciparum malaria in children, are equally typical for severe sepsis. Leukocytosis does not allow for discrimination either, as it has been described in up to 20% of young children with severe malaria.^14^,^15^ This can be (partially) explained by the activation of the same cytokine pathways in both conditions: Releasing debris from both, parasites and erythrocytes, including the so called malaria toxin glycosylphosphatidylinositol as well as malarial pigment (haemozoin) leads to the activation of peripheral blood mononuclear cells and consequently kicks off the cascade of pro-inflammatory cytokines which probably determines disease severity.^6^ Last but not least bacteraemia may complicate malaria in up to 8% of severe cases, especially in younger patients, increasing the risk for fatal outcome.^16^

Since severe malaria is a multisystem, multi-organ disease, children frequently present with a combination of the classical clinical phenotypes: cerebral malaria (CM), severe malarial anaemia (SMA), respiratory distress, and hypoglycaemia.

The former two, CM and SMA, are the most common complications of malaria in children. Cerebral malaria is defined by WHO as unrousable coma in a patient with *P.falciparum* parasitaemia in who other causes of encephalopathy have been excluded. Children with CM may develop focal neurological signs, decerebrated or decorticated posturing due to raised intracranial pressure, decreased level of consciousness or coma, behavioural changes, hallucinations, and seizures. Seizures can be protracted or multiple and may be followed by a long postictal state or they may be difficult to recognize if they present only by conjugate eye deviation, nystagmus, oral automatisms, salivation, and hypoventilation. Although most children with CM regain consciousness within 48 h and seem to make a full neurological recovery, approximately 20% die and up to 10% have persistent neurological sequelae.^6^ These are particularly associated with protracted or multiple seizures which may cause cognitive deficiency and/or epilepsy.

Malarial retinopathy (retinal abnormalities consisting of two unique features - patchy retinal whitening and focal changes of vessel colour) is highly specific,^17^ so not necessary^18^ for malarial encephalopathy not only in children.

Increasing evidence shows involvement of the angiopoietin-Tie-2 with retinopathy and mortality in paediatric cerebral malaria.^19^

Severe malarial anaemia (defined as haemoglobin concentration < 5 g/dl in the presence of *P.falciparum* parasitaemia) is more common in children than in adults. While mortality of SMA is low in asymptomatic children (approx. 1%), the presence of respiratory distress and metabolic acidosis is often (up to 30%) associated with a fatal outcome. According to the world malaria report 2011, the fatality rate for high risk populations approaches 40%.

The role of iron supplementation in the prevention and treatment of anaemia in malaria-endemic regions has been much debated. Iron deficiency has an adverse effect on child health, cognitive development and overall survival, and WHO guidelines thus recommend routine iron supplementation for children aged 6 months to 24 months living in areas where anaemia prevalence is 40% or more. Alterations of iron metabolism in the human host are, however, thought to increase resistance to infection by restricting the availability of iron to microorganisms. With effective malaria control, iron supplementation should not be withheld from children with anaemia in endemic areas.

Not only does the severity of malaria infection change with age, but the clinical manifestation of disease does as well: CM occurs more often in children aged 3 to 6 years; SMA is most likely to develop in children younger than 2 years. CM is more often associated with dehydration, hypoglycaemia, acidosis and respiratory distress; SMA is more often associated with spleen and liver enlargement. However due to it’s more subtle onset with less dramatic clinical manifestations than CM, this condition is often overlooked by the care giver in the initial phase and appropriate management of the malaria attack is delayed. SMA and CM account for most of all malaria-related deaths.^12^ In a study on severe malaria conducted in a urban reference Hospital in Bamako, Mali,^20^ the case fatality rate was 12% in children with CM only as compared to 2% in those with SMA only. Half of the deaths occurred within 12 hours of admission, 92% within 48 hours: these findings underscore the importance of early and precise diagnosis for efficient management of life-threatening malaria. Among the children who survived CM, 6,8% suffered from neurologic sequelae at day 45.

Respiratory distress (deep breathing, Kussmaul's respiration) is a clinical sign of metabolic acidosis, and has emerged as a powerful independent predictor of fatal outcome in falciparum malaria. It can be misinterpreted as cardiac failure and circulatory overload, especially if associated with severe tachycardia.

Blood lactate values are thus among the most useful predictors for stratifying the risk of fatality in children. However, measurement of blood lactate values requires specialized and costly equipment seldom available in African health centers.^18^

Hypoglycaemia (blood glucose concentration < 2.2 mmol/l) is also associated with a poor outcome in children with malaria as in other severe childhood infections. Clinical evidence suggests that hypoglycaemia in African children with severe malaria results from impaired hepatic gluconeogenesis rather than from quinine-induced hyperinsulinaemia^6^ but quinine therapy may increase the risk.

The kidneys may also be affected by, among others blackwater fever (massive haemolysis with haemoglobinuria and renal failure; a clinical picture seen more often in adult patients than in children), acute tubular necrosis, and immune complex glomerulonephritis (most characteristic for infections due to *P.malariae*).

Other complications less frequent in children are: pulmonary oedema, hepatic dysfunction, splenic rupture (a late complication, usually of vivax malaria, especially in those who have recently had their first infection), spontaneous bleeding, disseminated intravascular coagulation, hypotension, cardiovascular shock and multi-organ failure.

Tropical splenomegaly syndrome, also known as hyperreactive malarial syndrome, is rarely seen in children, as it usually occurs after repeat exposure to malaria. It is characterized by gross splenomegaly, high antibody levels of Plasmodium species, hypergammaglobulinaemia (mainly IgM), clinical and immunological response, and regression of splenomegaly over several months after antimalarial therapy.^11^

### Congenital Malaria

Pregnant women are more likely than others to be inoculated with and infected by malaria parasites and are more prone to severe forms, making adverse outcomes particularly common in primigravida women and their offspring.

Besides the mother, Malaria can infect also the placenta and the fetus, leading to low birth weight through intrauterine growth retardation and/or prematurity. Estimates for malaria induced low birth weight range from 7.8–45.3 of every 1000 live births and the associated mortality risks during the first month of life is about 40 times that of babies with normal birth weight.^21^

The parasite concentration dependent correlation between maternal peripheral blood parasitaemia, placental, and umbilical cord-blood (fetal) parasitaemia has long been known.^22^

All four types of human malaria can be transmitted congenitally, but the disease most often is associated with *P.vivax*. That congenital malaria is not seen more frequently is due in part to the effective barrier function of the placenta. Although congenital malaria develops in 0.1% of immune and 10% of no immune mothers in endemic areas, placental infection occurs in as many as one third of pregnant women. In endemic areas, distinguishing malaria acquired congenitally from that acquired by post-natal transmission from mosquitoes is difficult.

The onset of symptoms is insidious and usually occurs at 2 to 8 weeks of age. The typical malaria paroxysm is usually absent, with the infant presenting instead more sepsis like symptoms:^23^ irritability, poor feeding, vomiting and diarrhoea. Fever and hepatosplenomegaly may be found on physical examination. The most common laboratory finding is anaemia, but thrombocytopenia and (unspecific) hyerbilirubinaemia are also common. Therapy for the infected species of malaria is curative, but in contrast to the mother, the infant does not need treatment of the exo-erythrocytic stages of the parasite.

Interestingly, new evidence suggests that a subset of those vertically affected infants is also at higher risk of malaria infections later in life.^24^

Imported Malaria occurs in children in many non-endemic countries. Over 1000 imported cases in children are diagnosed in Europe each year.^25^ Returning to country of origin to visit friends and relatives is the main risk factor. Over three quarters of these individuals did not take the recommended malarial chemoprophylaxis for the region to which they were travelling. The diagnosis often is delayed because of lack of consideration of malaria as a cause of illness and unfamiliarity with the disease. In children with acquired immunity, the signs and symptoms of disease may be subtle and nonspecific, but fever is universal. The diagnosis of malaria should be considered in every child with fever or a history of recent fever who has visited a malaria-endemic area, irrespective of antimalarial prophylactic.

The differential diagnosis of fever in a patient with recent international travel history is broad. Common causes of fever by symptom complex are indicated in Table 2.

Although headache and fever may be severe in malaria, there is usually no neck stiffness or photophobia resembling that in meningitis. Although myalgias may be prominent in older children, they are not usually as severe as in dengue fever, and the muscles are not tender as in leptospirosis or typhus. In addition, malaria is not usually associated with a rash, helping to distinguish malaria from meningococcemia, typhus, leptospirosis, Rocky Mountain spotted fever, enteric fever, dengue fever, and viral hemorrhagic fevers. However, malaria may also coexist with other infections. The importance of malaria is underscored by a recent analysis from the GeoSentinel surveillance network: malaria was found to be the illness with the highest hospitalization rate (69%) in children after international travel.^26^

## Laboratory Findings

Haematological and biochemical parameters are often abnormal: normochromic normocytic anaemia occurs in 31–100% of cases, although the need for transfusion is rare (<2%). Thrombocytopenia (platelet counts below 150 × 10^9^/L) is characteristic of malaria and occurs in 50–70% but, unlike adults, usually is not associated with bleeding, even at very low counts. However, severe infections may be accompanied by prolonged coagulation profiles. Thrombocytopenia is associated with high parasitaemia levels, lower age, low Hb levels, increased MPV and platelet aggregate flag.^27^

The leukocyte count is usually low (mostly due to low lymphocytes and eosinophils) to normal, but it may be increased (especially neutrophils and monocytes) in severe infections. In fact leukocytosis was strongly found to be associated with younger age, deep breathing, severe anaemia, thrombocytopenia and death - irrespective of bacteraemia.^28^

Jaundice (30–50%) and raised liver enzymes (25–40%) are also relatively common but not usually associated with an adverse outcome.

In severe malaria, metabolic (lactic) acidosis, hypoglycaemia, hyponatraemia, and hypocalcaemia may be presenting. Hypoglycaemia occurs frequently with falciparum malaria: it's thought to occur as a result of parasite consumption of glucose, inadequate hepatic gluconeogenesis, and treatment with quinine. When present in children before treatment, hypoglycaemia is associated with a poor prognosis. Hyponatraemia may occur as part of the syndrome of inappropriate secretion of antidiuretic hormone in some patients. Serum creatinine and blood urea nitrogen may be elevated transiently or may rise significantly with acute renal failure. Malaria stimulates a polyclonal increase of immunoglobulins associated with rapid production of malaria-specific antibodies and reduced complements levels. CSF is usually normal.

## Diagnosis

WHO guidelines recommend prompt and accurate parasitological confirmation of malaria diagnosis by optic microscopy or rapid diagnostic tests based on lateral flow immunochromatography as part of an effective disease management,^29^ as delays in diagnosis are associated with an increased risk of severe malaria, requirement for intensive care and death.

As in adults, the gold-standard diagnosis of malaria rests on demonstration of the parasite in peripheral blood smears of a febrile child. Both thick and thin blood smears should be examined: the thick smear has the advantage of concentrating the parasites and thus increasing diagnostic sensitivity. The thin smear allows for positive identification of the malaria species.

In malaria endemic regions, there is a tendency to treat all fevers as malaria, particularly in high risk groups such as young children. As a result it is not uncommon for malaria to be clinically diagnosed and treated without microscopic confirmation or despite a negative blood smear.

The results of a retrospective study conducted in Uganda among children up to 15 years of age, with a diagnosis of malaria, provide evidence that children who did not have microscopy performed or had a negative blood smear had a higher risk of death than those with a positive blood smear. The higher mortality in children diagnosed and treated for malaria without microscopic confirmation is likely due at least in part to misdiagnosis and a lack of treatment for conditions other than malaria. These results argue for microscopy or rapid diagnostic testing of all children admitted with a presumptive diagnosis of malaria and evaluation of other causes of disease in children with negative results.^12^

In addition, in falciparum malaria, the percentage of parasitized red blood cells, the presence of *P.falciparum* schizonts and pigment deposits in peripheral polymorphonuclear leukocytes may indicate severe malaria.^11^

Serological tests provide confirmation of past malaria infection, but they don't help in the diagnosis of acute infections for treatment purposes. Furthermore, due to persisting maternal antibodies, their use in infants and young children is even more limited.

Additional diagnostic tests for malaria include but are not limited to rapid dipstick tests and polymerase chain reaction. There are now commercially available whole-blood rapid dipstick tests for falciparum malaria that are based on the qualitative detection of the histidine-rich protein 2 antigen of *P.falciparum*; however, their sensitivity and specificity depend on the pre-test probability making it difficult to express clear recommendations. In a recent study from Burkina Faso,designed to evaluate the accuracy of a rapid diagnostic test on the diagnosis of malaria infection and of malaria-attributable fever during low and high transmission season, the overall performance of the sensitivity of the test was below the WHO-recommended threshold of 95%. During the rainy season, almost 90% of febrile children below 1 year, and almost 85% of those between 1 and 4 years, had a positive rapid test, but a positive malaria test result had a Positive Predictive Value of only 82% and 69%, respectively.

Unfortunately even a negative test result could not rule out malaria in either group, with a residual probability of 16% and 10%, respectively. This raises concern especially for infants between 6–11 months, because, even at very low parasite densities (the most likely to go undetected by the rapid diagnostic tests), fever is almost invariably attributable to malaria.^30^

Although polymerase chain reaction techniques for the detection of malaria have good sensitivity and specificity, they are technically more complex, and require sophisticated and expensive equipment, so they are usually performed in reference laboratories and reserved for *a posteriori* diagnosis and epidemiological research.^10^

## Management and Treatment

### Uncomplicated Malaria

Treatment of malaria depends on the (presumptive) identification of the species of Plasmodium causing the infection, knowledge of the presence of resistant organisms in the area in which the malaria was contracted, national guidelines, antimalarial availability, individual patient factors and whether the malarial illness is categorized as either uncomplicated or severe.

For falciparum malaria, the urgent initiation of appropriate therapy is especially critical, because *P.falciparum* infections can cause rapidly progressive illness and death.

In endemic areas children with uncomplicated malaria, low parasitaemia, no vomiting and who maintain their nutrition and hydration orally may be treated with oral antimalarials, on an outpatient basis.^10^

Where possible and feasible, a thick and thin blood smear, FBCs, electrolytes, blood glucose, and renal and liver function tests should be performed on all patients hospitalized, as well as testing for glucose-6-phosphate dehydrogenase (G6PD) deficiency, if *P.vivax* can not be excluded.^11^

Drug combinations, rather than monotherapy, are now seen to be the best solution for treating malaria, but the primary problem with using drug combinations in Africa is cost: in much of the continent people have malaria several times a year, and treatment cost – including procurement of drugs - could be prohibitive both for governments and households.^31^ This is one of the reasons why too many cases in Africa are still treated with monotherapy with a high risk of treatment failure.^13^

Artemisinin and its derivatives are now a standard component, due to their high plasmodium killing rates and their capacity to target both, sexual and asexual stages they prevent both, clinical deterioration and transmission. Combination of an artemisinin derivative with a long-acting antimalarial drug reduces treatment duration to only 3 days. Artemisinins are generally safe and well tolerated, and are recommended by WHO as first-line treatment for *P. falciparum* and chloroquine-resistant *P.vivax* infection. The main artemisinin-based combination therapies (ACTs) are artesunate combined with either mefloquine or amodiaquine, artemether combined with lumefantrine, and dihydroartemisinin with piperaquine. The artemisinin derivatives are safe and well tolerated by young children, and so the choice of ACTs will be determined largely by the safety and tolerability of the partner drug.

Sulfadoxine-pyrimethamine should be avoided in the first weeks of life because it competitively displaces bilirubin with the potential to aggravate neonatal hyperbilibinaemia. Furthermore the correct dosing in young children still needs to be defined [32]. Primaquine should also be avoided in the first month and in children known to have severe G6PD deficiency.

Alternatives such as clindamycin and doxycycline may also be given, but only in children >8 years because of risk of dental hypoplasia and permanent teeth discoloration. With these exceptions there is no evidence for specific serious toxicity for any of the other currently recommended antimalarial treatments in infancy.^29^

Chloroquine remains recommended for the treatment of infections caused by *P.malariae, P.ovale* and *P.knowlesi*, also ACT seem to be equally effective.

In *P.vivax* and *P.ovale* infections, patients recovered from the first episode of illness may have additional attacks, or relapses, after months or even years without symptoms, because these Plasmodium species have dormant liver stage parasites (hypnozoites) that may reactivate. Treatment with primaquine phosphate for 14 days should be included in the treatment of the first attack to eradicate the hepatic hypnozoites.

Of course, HIV-infected children should receive prompt, effective antimalarial treatment according to the WHO guidelines. While there is evidence that Lopinavir/ritonavir based antiretroviral treatment can lower the risk for malaria in children^33^ other combinations may be associated with a higher incidence of neutropenia (artesunate and amodiaquine)^34^ or hepatotoxicity (artesunate and amodiaquine plus efavirenz).^35^

With regard to paracetamol use, current WHO guidelines on the management of fever recommend it’s use in children with a temperature of 38.5°C or above. However, in a recent study there seems to be no statistically significant difference in temperature or other symptoms like vomiting or headache between children receiving paracetamol or placebo.^36^

### Severe Malaria

As already said earlier, the risk of death from severe malaria is greatest in the first 24 hours.

Patients with severe malaria infections or those unable to take oral medications should thus be hospitalized and managed as any emergency: airway patency should be checked, and oxygen should be given. Intravenous access should be rapidly established to allow for lab work (including blood cultures when possible) and parenteral antimalarial therapy.

If referral to a treatment facility able to administer IV treatment cannot be accomplished within 6 h, pre-referral treatment with intramuscular artesunate,^37^ artemether, or quinine, or rectal artesunate (10 mg/kg BW single dose) is recommended. The patient should than be referred to a facility where complete parenteral treatment can be given. Only if referral is impossible, rectal treatment should be continued until the patient can tolerate oral medication to be than continued with a full course of the recommended ACT for uncomplicated malaria.^38^

Indications for hospitalization include cerebral malaria, severe anaemia, haemoglobinuria, renal failure, pulmonary oedema, coagulopathy, severe thrombocytopenia, shock, high parasitaemia, metabolic acidosis, hypoglycaemia, intractable vomiting, dehydration, seizures, or altered level of consciousness.^10^ Among them, respiratory distress and impaired consciousness are indicators of a poor prognosis that should trigger immediate parenteral antimalarial treatment with any effective antimalarial first available. So eventually less efficacious as in non-malaria associated seizures,^39^ intravenous diazepam should be administered for any seizure lasting more than 5 minutes.

For parenteral treatment of severe malaria the cinchona alkaloids (quinine and – in the US quinidine) and the artemisinin derivatives (artesunate, artemether and artemotil) can be used.^38^ However, recent evidence from the AQUAMAT trial,^40^ a multi-centre study conducted in African children hospitalized with severe malaria, showed a significant mortality reduction by 22.5% in the artesunate group when compared to the quinine group. The superiority of parenteral artesunate over quinine for the treatment of severe malaria in both adults and children and in different regions of the world is reflected also in the latest Cochrane Review.^41^

Artesunate seems to offer additional advantages, ranging from ease of administration (no cardiac monitoring) to the reduction in the incidence of convulsions, coma, and hypoglycaemia developing after discharge.

For children, artesunate 2.4 mg/kg BW IV or IM given on admission (time = 0), then at 12 h and 24 h, then once a day is the recommended treatment. Artemether, or quinine,is an acceptable alternative if parenteral artesunate is not available: artemether 3.2 mg/kg BW im given on admission then 1.6 mg/kg BW per day; or quinine 20 mg salt/kg BW on admission (IV infusion or divided im injection), then 10 mg/kg BW every 8 h; infusion rate should not exceed 5 mg salt/ kg BW per hour.

Children with anaemia associated with severe malaria may require blood transfusion. Platelet transfusions for thrombocytopenia are generally not recommended because thrombocytopenia is not associated with bleeding problems in children.

Blood glucose should be monitored every 4 h and haemoglobin and parasite count at least daily.

Empirical parenteral antibiotic treatment with a third-generation cephalosporin or even a quinolone should also be given as co-infection with malaria and (multidrug resistant) gram-negative bacteria, like non-typhoidal salmonellae,^42^ are more frequent than previously thought and may not always be identified by current WHO guidelines.^43^

Regarding the duration of the antimalarial treatment, currently experts recommend to give parenteral antimalarials in the treatment of severe malaria for a minimum of 24 h, once started or until the patient is about to tolerate oral medication , before giving the oral follow-up treatment, which should consist in a full course of an effective ACT (artesunate plus amodiaquine or artemether plus lumefantrine or dihydroartemisinin plus piperaquine) or artesunate (plus sulfadoxine-pyrimethamine, or clindamycin or doxycycline) or quinine (plus clindamycin or doxycycline). Where available, clindamycin may be substituted in children and pregnant women; doxycycline cannot be given to these groups. Regimens containing mefloquine should be avoided, if the patient presented initially with impaired consciousness, because of an increased incidence of neuropsychiatric complications associated with mefloquine, not only following cerebral malaria.^44^

With regard to supportive treatment, bolus-fluid resuscitation should be used with caution. Recent evidence from Africa shows that fluid-bolus with either albumin or saline, as compared with control, increased the absolute risk of death for children with severe febrile illness and impaired perfusion at 48 hours by 3.3 percentage points and the risk of death, neurologic sequelae, or both at 4 weeks by nearly 4 percentage points. The risk was higher for children with a positive malaria test as compared to those with a negative malaria test and for those with <5g/dL haemoglobin as compared to those with higher haemoglobin levels.^45^ As fluid boluses can dilute haemoglobin, reduce tissue oxygen delivery, or lead to cardiac failure, and high levels of circulating antidiuretic hormone (ADH) will reduce fluid requirements in meningitis or pneumonia, this practice may be dangerous in children with malaria.^46^ This may however not hold true for settings in highly developed countries, where Paediatric Critical Care Medicine including Paediatric Advanced Life Support Systems are readily available.

## Prevention

The two components of malaria prevention are reducing exposure to infected mosquitoes and chemoprophylaxis.

### Chemoprophylaxis

All children (including immigrants and those traveling to a malaria-endemic region to visit friends and relatives) should take an appropriate antimalarial drug. The choice of which chemoprophylactic agent to use should be based on the presence of chloroquine-resistant or mefloquine-resistant strains in the specific area.

Intermittent administration of a full therapeutic dose of an antimalarial drug (or a combination of drugs) at specified timepoints, whether or not parasites are present is known as Intermittent Preventive Treatment (IPT). IPT shows great benefit in areas of high transmission and for specific risk groups (infants, high risk children and/or pregnant women). Sulfadoxine-pyrimethamine is particularly suited for this approach as it has a long half-life. An analysis of pooled efficacy data from six randomized trials of sulfadoxine-pyrimethamine given to infants showed that compared with placebo, IPT had a protective efficacy of 30.3% against clinical malaria, and 38.1% against malaria associated hospital admissions, but no efficacy on mortality.^47^ We have used this strategy in Burkina Faso in a cohort of HIV-infected children not receiving co-trimoxazole with good results (unpublished data) and found it equally effective as co-trimoxazole. Unfortunately, the spread of sulfadoxine-pyrimethamine resistance is rapidly compromising its future effectiveness.

A further development of IPT is Seasonal Malaria Chemoprevention (SMC). WHO is now recommending SMC for the prevention of falciparum malaria among children less than 5 years of age in areas with highly seasonal malaria transmission as the Sahel sub-region. A complete 3-day treatment course of amodiaquine plus sulfadoxine-pyrimethamine (AQ+SP) should be given to children aged between 3 and 59 months at monthly intervals, to a maximum of four doses during the malaria transmission season. SMC should not be given to children with severe acute illness or unable to take oral medication, to HIV-positive patients on co-trimoxazole, to a child who has received a dose of either AQ or SP drug during the past month or who is allergic to either drug. The recommendation is based on results from 7 studies on SMC (IPTc) showing a reduction of 75% in malaria episodes in children less than 5 years of age without significant side effects.^48^

### Vector Control

Key interventions currently recommended by WHO for the control of malaria in endemic areas are the use of insecticide treated nets (ITNs) and/or indoor residual spraying (IRS) for vector control, and prompt access to diagnostic testing of suspected malaria and treatment of confirmed cases.

Community randomised trials in Africa have shown that full coverage with insecticide-treated nets can halve the number of episodes of clinical malaria and reduce all-cause mortality in children younger than 5 years of age. Initial fears that reducing malaria transmission might paradoxically increase child mortality through delayed acquisition of malarial immunity have not been realised. When used by pregnant women, insecticide-treated nets can lead to substantial reductions in low birth weight, placental parasitaemia, stillbirths, and miscarriages. The proportion of households in Africa estimated to own at least one insecticide-treated net rose from 17% in 2006 to 50% in 2011, with 24% of children younger than 5 years of age using an insecticide-treated net during 2008.

### Repellents

Use of topical insect repellent is an important component of the prophylaxis against arthropod bite vector borne diseases too. Rational repellent prescription for a child must take into account age, active substance concentration, topical substance tolerance, nature and surface of the skin to protect, number of daily applications, and the length of use in a benefit-risk ratio assessment perspective. The repellents currently recommended in the 2012 edition of the Yellow Book^50^ comprise: **1) DEET** (chemical name: N,N-diethyl-m-toluamide or N,N-diethyl-3-methyl-benzamide). **2) Picaridin** KBR 3023 or Icaridin (chemical name: 2-(2-hydroxyethyl)-1-piperidinecarboxylic acid 1-methylpropyl ester). **3) Oil of lemon eucalyptus** (OLE) or **PMD** (chemical name: para-menthane-3,8-diol) commonly known as Citriodiol (a mixture of cis and trans para-menthane-3,8-diol). **4) IR3535** (chemical name: 3-[N-butyl-N-acetyl]-aminopropionic acid, ethyl ester).

A table comparing these substances in detail has been published by F. Sorge.^51^

Efficacy and duration of protection for all these products are markedly affected by ambient temperature, amount of perspiration, exposure to water, abrasive removal, etc. In general, higher concentrations of active ingredients provide longer duration of protection, although DEET efficacy seems to reach a plateau between 30 - 50% covering > 6 hours. The American Academy of Pediatrics (AAP) recommends that repellents should contain *no more than 30% DEET* when used on children.^52^

In field trials the minimum concentration of each of the four agents required to be effective for 3 hours against most arthropods is 20% (independent of the application form as cream, roll-on or spray), products with <10% active ingredient may offer protection only for 1 or 2 hours.

The common side effects of these agents described so far are usually mild local irritative dermatitis and allergy. Prolonged exposure to DEET has been related to more severe side effects, namely neurotoxicity. A comprehensive evaluation of the DEET health effects on humans including the rare reports of adverse effects associated with dermal application in children, can be found on the ATSDR website.^53^

The European Parliament amended its directive on biocide products in 2010,^54^ including DEET among the products intended for direct application to human skin. The directive requires a detailed label, showing instructions including amount and frequency of application as well as a deterrent for ingestion. Furthermore the following age restriction for DEET containing products is mentioned: should not be used on children less than two years old, and use should be restricted for children between two and twelve years old, except where motivated by the risk for human health through e.g. outbreaks of insect-borne diseases.

However, national recommendations on the use of DEET in children differ in the lower age limit, with the Canadian Recommendation not giving any age restriction,^55^ the British Health Protection Agency (HPA)^56^ as well as the AAP^52^ allowing to begin at 2 months of age and information made available by the German Ministry^57^ waiting until 3 months of age. In Italy there is no national recommendation, but regional guidelines^58^ advise to start treatment no earlier than age 12 years.

The most detailed guideline (with respect to age classes and number of daily applications) has been published by the Groupe de pédiatrie tropicale of the Société française de pédiatrie,^59^ and has been confirmed recently by the relevant French Scientific Societies,^60^ giving yet another lower age limit: Newborns and infants < 6 months: avoid all repellent use. Products containing (P)Icaridine should be avoided in children <24 months. Products containing OLE specify that they should not be used on children aged <3 years. Infants 3 to 12 months: only in circumstances of exceptional exposure use DEET (20–30%) not more than once daily. From ages 1 to 12 years, 2 daily DEET applications at the same concentration may be safely used; 12 years old through adulthood: 3 applications daily. However, DEET is not recommended for children with a history of seizures and for pregnant and lactating women, because of its potential neurotoxicity on the fetus and newborn.

Citriodiol (at 20–30%) and Ir3535 (20%) may be safely used once daily in children between 6 – 12 months and twice daily between 12 months and 12 years. Above 24 months IR3535 may be used at a concentration as high as 35%. For children aged more than 12 years, adult guidelines apply.

International recommendations regarding the use of topical repellents in children for the prophylaxis of arthropod borne diseases are limited to short/medium term usage (several weeks). But the use of repellents is prolonged or chronic among the majority of children living in subtropical regions where these vector borne diseases are endemic. The toxicity of topical repellents used continuously for longer time periods has not been assessed in pediatric age groups. So if the lenght of expected exposure is over 3 months, other preventive measures are to be preferred.

Whatever repellent is used, once returned in a safe indoor environment, don’t forget to wash the skin with soap and water to remove any repellent. Do not spray repellent directly in the face, nor on cuts, wounds or otherwise irritated skin. Furthermore use of products that combine repellent (DEET) with sunscreen is discouraged, as sunscreen should be applied first (and may be repeated).

Clothing, hats, shoes, bed nets, mesh jackets, and camping gear can be treated with permethrin for added protection. Make sure infants and children don’t put treated items in their mouth!

All newborns and infants in their first months are protected best from mosquitoes by using an infant carrier draped with mosquito netting with an elastic edge for a tight fit or make sure to tuck the bed net firmly under the mattress.

## Figures and Tables

**Table 1:** Age-specific clinical manifestations of malaria (adapted from)^10^

Congenital malaria	Infants and toddlers	Older children

Fever (without paroxysm)	Fever (irregular or continuous)	Fever (intermittent or continuous)
Irritability	Irritability	Chills/rigor
Listlessness	Anaemia	Diaphoresis
Anaemia	Poor feeding	Headache
Jaundice	Vomiting	Backache
Poor feeding	Lethargy	Myalgia
Vomiting	Jaundice	Abdominal pain
Regurgitation	Splenomegaly	Nausea/vomiting/diarrhea
Loose stools	Hepatosplenomegaly	Fatigue
Splenomegaly		Anaemia
Hepatosplenomegaly		Seizures
Cyanosis (in severe cases)		Decreased mental status
		Hepatosplenomegaly

**Table 2:** Differential diagnosis of malaria (adapted from)^10^

**Intermittent fevers:** borreliosis, brucellosis, trypanosomiasis, kala azar (visceral leishmanasis), babesiosis, sequential common infections, mononucleosis, rat-bite fever, idiopathic periodic fever, familial Mediterranean fever, lymphoma, juvenile reumathoid arthritis
**Fever and headache:** meningitis, encephalitis, sinusitis, influenza, typhus, enteroviral infection, tuberculosis, pneumonia, occult bacteraemia, common viral infections
**Fever, abdominal pain, vomiting, diarrhoea, and malaise:** enteric (typhoid) fever, viral or bacterial gastroenteritis, hepatitis, pyelonephritis, schistosomiasis (Katayama fever), amebiasis
**Fever and jaundice with recent tropical travel:** dengue fever, viral hemorrhagic fever, leptospirosis, yellow fever, plague, hepatitis, enteric (typhoid) fever, typhus, haemolytic- uremic syndrome (Shigella, E.coli)
